# Sedimentation Pulse in the NE Gulf of Mexico following the 2010 DWH Blowout

**DOI:** 10.1371/journal.pone.0132341

**Published:** 2015-07-14

**Authors:** Gregg R. Brooks, Rebekka A. Larson, Patrick T. Schwing, Isabel Romero, Christopher Moore, Gert-Jan Reichart, Tom Jilbert, Jeff P. Chanton, David W. Hastings, Will A. Overholt, Kala P. Marks, Joel E. Kostka, Charles W. Holmes, David Hollander

**Affiliations:** 1 Department of Marine Science, Eckerd College, Saint Petersburg, FL, United States of America; 2 College of Marine Science, University of South Florida, Saint Petersburg, FL, United States of America; 3 Department of Earth Science, Utrecht University, Utrecht, The Netherlands; 4 Marine Geology Department, Royal Netherlands Institute for Sea Research, Texel, The Netherlands; 5 Department of Earth, Ocean & Atmospheric Science, Florida State University, Tallahassee, FL, United States of America; 6 Schools of Biology, Georgia Institute of Technology, 310 Ferst Drive, Atlanta, Georgia 30332–0230, United States of America; 7 Schools of Earth & Atmospheric Sciences, Georgia Institute of Technology, 310 Ferst Drive, Atlanta, Georgia, 30332–0230, United States of America; 8 Environchron, 9103 64th Ave. E., Bradenton, FL, United States of America; University of California, Merced, UNITED STATES

## Abstract

The objective of this study was to investigate the impacts of the Deepwater Horizon (DWH) oil discharge at the seafloor as recorded in bottom sediments of the DeSoto Canyon region in the northeastern Gulf of Mexico. Through a close coupling of sedimentological, geochemical, and biological approaches, multiple independent lines of evidence from 11 sites sampled in November/December 2010 revealed that the upper ~1 cm depth interval is distinct from underlying sediments and results indicate that particles originated at the sea surface. Consistent dissimilarities in grain size over the surficial ~1 cm of sediments correspond to excess ^234^Th depths, which indicates a lack of vertical mixing (bioturbation), suggesting the entire layer was deposited within a 4–5 month period. Further, a time series from four deep-sea sites sampled up to three additional times over the following two years revealed that excess ^234^Th depths, accumulation rates, and ^234^Th inventories decreased rapidly, within a few to several months after initial coring. The interpretation of a rapid sedimentation pulse is corroborated by stratification in solid phase Mn, which is linked to diagenesis and redox change, and the dramatic decrease in benthic formanifera density that was recorded in surficial sediments. Results are consistent with a brief depositional pulse that was also reported in previous studies of sediments, and marine snow formation in surface waters closer to the wellhead during the summer and fall of 2010. Although sediment input from the Mississippi River and advective transport may influence sedimentation on the seafloor in the DeSoto Canyon region, we conclude based on multidisciplinary evidence that the sedimentation pulse in late 2010 is the product of marine snow formation and is likely linked to the DWH discharge.

## Introduction

The 2010 Deepwater Horizon (DWH) blowout event discharged >600 million L of oil and large quantities of natural gas (e.g., methane, ethane, butane, propane) into NE Gulf of Mexico (GoM) waters over an ~3-month period [1–4]. In addition, almost 7 million L of chemical dispersants were injected into the deep-sea environment for the first time at such a great depth (~1500 m) [5–7]. It is estimated that at least 60% of the oil released reached the sea surface where it was subjected to a variety of processes including biotic and abiotic reactions, cleanup activities, transport out of the study area or to nearby beaches by physical processes, evaporation, and settling to the sea floor [5, 7, 8]. The remaining ~40% of the oil and an unknown quantity of the deep injected dispersants never reached the surface and remain unaccounted for [5, 9].

**Fig 1:**
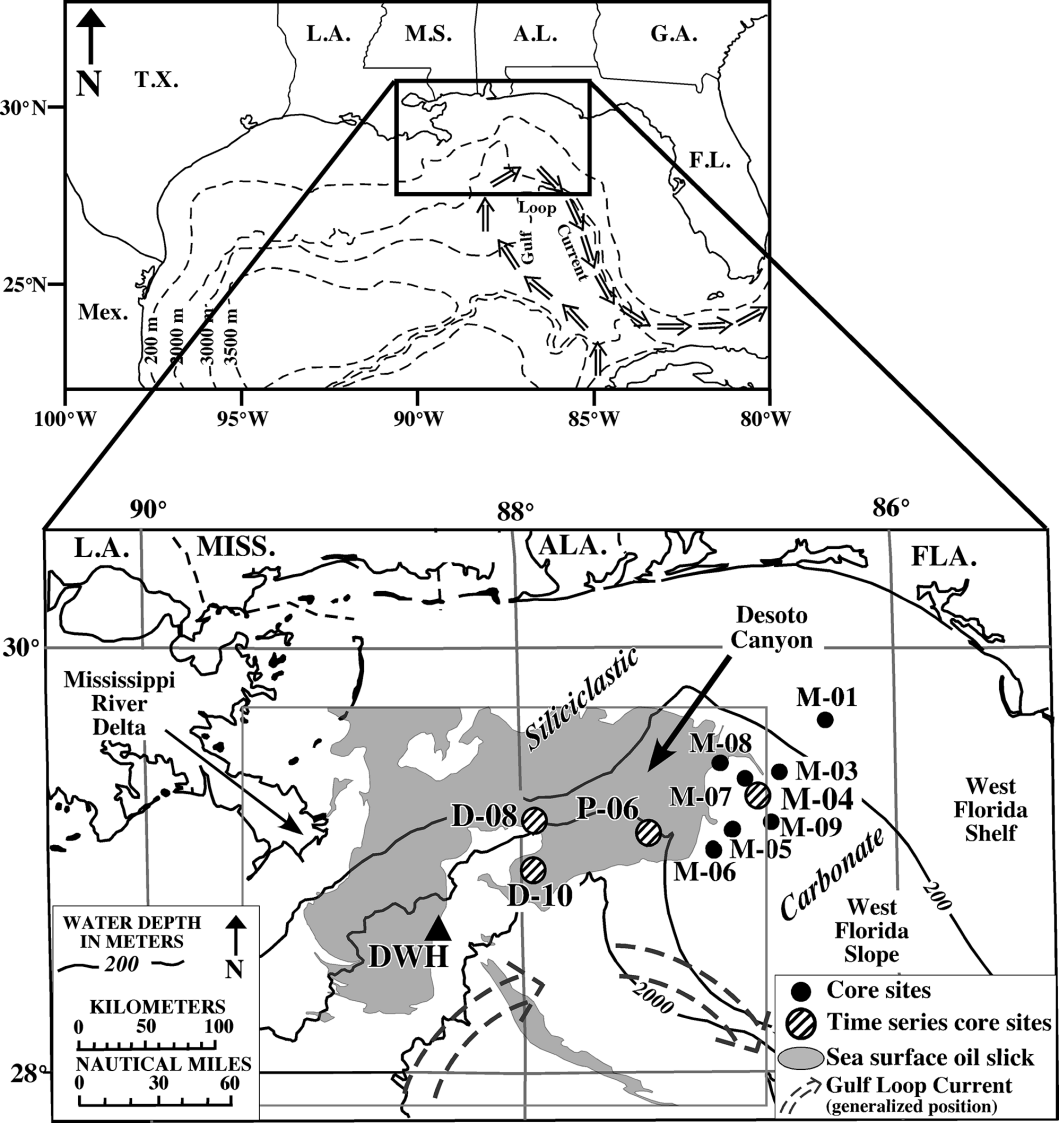
Study area map. Location map of the northeastern Gulf of Mexico showing core sites discussed here in proximity to the DWH wellhead, Desoto Canyon, the Mississippi River, and the extent of the sea surface oil slick (gray shading) mapped by Garcia-Pineda [11].

An oil slick was detected in open marine and coastal surface waters from Louisiana to Florida, including the DeSoto Canyon region (Fig 1) [10, 11]. Subsurface hydrocarbon-rich plumes were initially detected to the southwest of the wellhead at depths between ~1000 and 1200 m, with a more diffuse plume identified between ~50 and 500 m [1, 4, 6, 9, 12]. Later, subsurface plumes were identified between ~1000 and 1400 m, and ~400 m to the northeast of the wellhead, in the DeSoto Canyon region [13].

An unusually large marine snow event was documented in oil contaminated surface waters following the blowout [14]. The marine snow may have formed from extracellular polysaccharides and other exudates produced by phytoplankton and/or bacterioplankton in response to exposure from surfaced oil [15, 16]. Originally thought to have formed *in situ* in direct response to the oil [14], the marine snow was no longer present in surface waters by the end of June 2010, likely due to rapid sedimentation to depth [15]. It was reported that as the marine snow attracted particulates on the sea surface and in the water column, it lost buoyancy and rapidly sank in what was termed a “dirty blizzard” [13, 17], potentially creating a sedimentation pulse on the sea floor [16–19]. The wide range in particle size and density within the marine snow was attributed to the heterogeneous nature of the particles. Approximately 60% of the marine snow particles fell between diatom and coccolithophore densities [14]. Calculated settling rates [14] suggest it took particles a few days to several weeks to reach the seafloor for the depth range of cores collected in this study. Sea floor sediment traps continued to accumulate an abnormally large amount of marine snow throughout the Fall 2010. A sediment trap deployed by Passow (Pers. Comm., 2013) ~120 m above the seafloor (~1400 m depth) in the vicinity of the DWH wellhead, began collecting samples in late August of 2010. The first cup (collecting until mid September, 2010) was described as “overflowing”, with more than 1.5 g/m^2^/d on average during the 3-week period. Sedimentation rates in September and early October of 2010 were 2–5 times higher than those observed one year later (U. Passow, Pers. Comm., 2013).

Previous marine snow investigations have documented that once the rapidly sinking marine snow reaches the sea floor, it may cover and suffocate benthic communities, potentially causing temporary anoxic bottom conditions [20–22]. Although little is known about marine snow formation at depth, it was suggested that marine snow may have formed within subsurface plumes as well [7, 14, 23].

Deep GoM sediment impacts following the DWH event have not been well documented, but a 3.8–5 cm-thick reddish-brown surface layer within 10 km of the DWH wellhead was interpreted as freshly sedimented material in response to the “dirty blizzard” [16, 17, 19]. The primary objective of this study was to investigate the impacts of the DWH discharge as recorded in bottom sediments from the DeSoto Canyon area approximately 20–100 nautical miles east/northeast of the DWH wellhead. Specific questions addressed include: 1) Did the event directly or indirectly alter the temporal and/or spatial sediment distribution patterns in the study area, and if so, how? 2) What is the sedimentary signature of the event, and how is it manifested in bottom sediments? and 3) What is the long-term preservation potential of the event signature in the sedimentary record?

### Setting

The study area is located along the NW Florida outer continental shelf and slope, to the east of the DWH wellhead, in ~100 m to >1500 m water depths (Fig 1). The most conspicuous physiographic feature in the study area is the DeSoto Canyon, an S-shaped submarine canyon located ~100 km south of the Florida panhandle. The canyon exhibits both erosional and depositional features and is constrained by at least five salt domes [24].

Bottom sediments surrounding the DeSoto Canyon are complex in both texture and composition, reflecting the different sedimentologic regimes to the west and east. To the west, sedimentation is dictated by the Mississippi River and the input of siliciclastics into the NE GoM. Bottom sediments are dominated by quartz sand on the shelf forming the “MAFLA” (Mississippi-Alabama-Florida) Sand Sheet [25, 26]. Slope sediments are siliciclastic-rich silts and clays, with pelagic carbonate oozes making up a larger fraction in deeper regions [27]. To the east, sedimentation is dictated by biogenic carbonate production forming the West Florida Sand Sheet on the mid-outer shelf, grading down slope into the finer-grained West Florida Lime Mud [26]. Sediment accumulation rates calculated from ^14^C dates range from ~17 cm/ky northwest of DeSoto Canyon [28] to ~10 cm/ky to the southeast [28, 29]. These are linear accumulation rates (LAR), which do not account for down-core compaction. A mass accumulation rate (MAR) of 0.05 g/cm^2^/yr was determined by ^210^Pb methods for a single core in the DeSoto Canyon region at ~1850 m water depth [30].

Typically, the highest proportion of carbonate in bottom sediments, frequently in excess of 75%, occurs on the west Florida shelf, and carbonate content decreases from ~60% at the shelf-slope break to ~25% at the base of slope [27]. To the west of the canyon, the carbonate content exhibits an opposing pattern with a basin-ward increase, likely reflecting a seaward decrease in Mississippi River influence and corresponding increase in pelagic carbonate deposition [27]. Particulate organic carbon (POC) for one core in the DeSoto Canyon region at ~1850 m water depth ranged from ~0.67%–1.17% for the upper 18.5 cm of the core [30]. Clay mineral assemblages in bottom sediments are also complex. In general, smectite is the dominant clay mineral west of the canyon, due to input from the Mississippi River, while kaolinite is dominant east of the canyon reflecting input from the Apalachicola River [27, 31, 32]. The differences in sediment types/sources on either side of Desoto Canyon makes this an ideal region to investigate if the DWH event altered natural sedimentation patterns/processes; as any alteration should be readily visible as a change in the relative abundance of the two sediment types.

## Methods

### Sample collection

Multicores were collected from seventeen sites in the DeSoto Canyon region of the NE Gulf of Mexico (GoM) during November/December 2010, using a MC-800 Multicorer capable of collecting up to eight, 10-cm diameter by 70 cm-long cores per deployment with minimal disturbance to the sediment-water interface (Fig 1). Eleven of these cores, collected 20–100 nautical miles (NM) northeast of the DWH wellhead from 100 m to >1500 m water depths, were chosen for detailed analyses based on the following criteria: 1) no visible evidence of a break in sediment deposition, 2) well preserved sediment-water interface, 3) no visible evidence of sediment mixing, 4) no evidence of gravity flow deposition, 5) representative coverage of different water depths (including the depths of the two documented subsurface plumes at ~400 m and 1000–1400 m), and 6) representative coverage of both the siliciclastic–dominated and carbonate–dominated sediment regimes west and east of DeSoto Canyon, respectively. Four of the eleven sites (M-04, P-06, D-08, D-10) were reoccupied and cored up to three more times over the following two years to obtain a temporal perspective, and are referred to here as ‘time series’ sites (Fig 1). No permissions were required for collection of cores at any sites and this study did not involve endangered or protected species.

One core per deployment was split longitudinally, photographed, and described visually. For select cores, the entire core half was x-rayed to ensure stratigraphic integrity and to detect subtle sedimentary structures. One core per deployment was extruded at 2–5 mm intervals for sediment texture/composition and geochronological analyses. The 2 mm sampling interval was focused on the surficial 2–10 cm (based on visual descriptions), which represents most recent deposition, and would ensure the greatest possible resolution of recently impacted sediments. A calibrated threaded rod attached to a tight fitting plunger was used to extrude the core vertically upward through a flat acrylic surface, where the sample was carefully extracted from the top. Once extruded, samples were weighed immediately to provide the wet weight required for determining pore water content. Each sample was then freeze-dried and weighed for dry weight to calculate dry bulk density.

### Sediment texture and composition

Sediment texture/composition analyses were conducted on all cores collected in November/December 2010, and included grain size, calcium carbonate content (%CaCO_3_), and total organic matter (%TOM). Grain size was determined by wet sieving the sample through a 63 μm screen. The fine-size (<63 μm) fraction was analyzed by pipette [33] to measure %silt/%clay. The sand-size (>63 μm) fraction was volumetrically too small to analyze further and is reported here as %sand. Carbonate content was determined by the acid leaching method according to Milliman [34]. Total organic matter (TOM) was determined by loss on ignition (LOI) at 550°C for at least 2.5 hours [35].

Additional compositional analyses were performed on a subset of the time series cores collected in November/December 2010, using a variety of techniques including microscopic (digital and SEM), energy dispersive x-ray (EDS), core-scanning x-ray fluorescence (XRF) and x-ray diffraction (XRD). Microscopic analysis was performed using a digital microscope (*Dino-Lite)* at magnifications ranging from 70x to 220x, and by Scanning Electron Microscope (SEM) at magnifications ranging from 2kx to 8kx. The latter was conducted on a Hitachi S-3500N SEM at the University of South Florida College of Marine Science, St. Petersburg, FL. Select grains identified under the SEM, were analyzed by EDS to determine the elemental composition.

Entire core elemental compositions were determined for all four 2010 time series cores at the mm-scale by XRF core scanning at the NIOZ (Royal Netherlands Institute for Sea Research) laboratory using standard optimized settings [36]. This technique provides a rapid, non-destructive means to analyze sediment cores at high-resolution for elemental composition. Analysis was performed on an Avaatec XRF Core-Scanner with a 1mm by 1cm wide slit window at 1mm step resolution. Whole sediment cores were covered with a thin film transparent to X-rays to prevent sediment sticking to the device and prevent the core from drying out. The analysis chamber was flushed with He to provide accurate measurement of light elements.

Select cores/samples were analyzed by XRD to determine mineralogical content. Samples were analyzed on a Bruker D-8 Advanced system using cobalt radiation at the University of Georgia Department of Geology.

### Microbial community structure

Based on radiocarbon evidence and proximity to the wellhead, microbial community structure was examined on 2010 time series core D-10 using next generation sequencing. Total genomic DNA was extracted from 0.5 g of sediment from each core section using a MoBio PowerSoil DNA extraction kit according to the manufacturer’s protocol (MoBio Laboratories, Carlsbad, CA). DNA concentration was determined using a Quant-IT kit (Life Technologies, Grand Isle, NY). DNA was sent to the Institute for Genomics and Systems Biology Next Generation Sequencing Core facility at Argonne National Laboratory for SSU rRNA gene sequencing. Sequencing reactions were conducted on an Illumina MiSeq platform in a 151x151x12 bp run using sequencing primers and procedures that were previously described [37]. The resulting sequences were processed using QIIME v.1.7 [38]. Briefly, reads were demultiplexed using QIIME default paramaters (reads were truncated if 3 consecutive bases had a phred score less than 3, only reads >114 bases were retained). Sequences that were less than 60% similarity to any sequence in the GreenGenes database (v.13-5) [39] were discarded. Operational taxonomic units (OTU) were defined at 97% similarity using UCLUST [40], and only OTUs that represented more than 0.005% of the total reads were considered [41]. Putative taxonomy was assigned to representative reads using RDP classifier [42, 43] at 50% confidence and all reads assigned to the sequences from predominant photosynthetic microbial groups, cyanobacteria and phytoplankton chloroplasts were extracted. Samples were grouped based on sediment depth (0–2 cm, > 2 cm depth intervals) and the relative abundance of phototroph sequences was transformed to meet assumptions of normality. A Welch’s t test was used to compare the two groups.

### Natural abundance radiocarbon

Subsamples of 2010 time series cores P-06 and D-08 and D-10 were prepared for Δ^14^C analysis at the National High Magnetic Laboratory at Florida State University. Dried sediment was acid treated in 10% HCl to remove carbonates then combusted and purified to CO_2_ following the methods of Choi and Wang [44]. The break seal tubes for Δ^14^C analysis were sent to National Ocean Sciences Accelerator Mass Spectrometry Facility (NOSAMS) where they were converted to graphite targets and analyzed by accelerator mass spectrometry [45]. Values are reported in the Δ^14^C notation according to Stuiver and Polach [46].

### Benthic foraminifera

Subsamples of 2010 time series cores P-06 and D-08 were freeze-dried, weighed and washed with a sodium hexametaphosphate solution through a 63-μm sieve to disaggregate the clay particles from foraminifera tests. The >63-μm fraction was dried, weighed again, and stored at room temperature. All benthic foraminifera were picked from the samples, identified, and counted. Foraminifera assemblage density values were reported in individuals per unit volume (indiv./cm^3^) [47]. The values were normalized to the known wet volume of each sample based on the diameter of the core tube (10 cm) and the height of each sample (2 or 5 mm).

### Biomarkers

Biomarkers were analyzed using a modified EPA method [48] for the analysis of biomarkers. Freeze-dried samples were extracted (at 100°C, 1500 psi, 9:1v:v dichloromethane: methanol) using an ASE system (Dionex 200). Previous to extraction, samples were spiked with d_50_-Tetracosane. Activated copper (40 mesh, 99.9%, Sigma-Aldrich, USA) was added and lipid extracts were clean using solid-phase extraction (SPE) with silica/cyanopropyl glass columns (SiO_2_/C_3_-CN, 1 g/0.5 g, 6 mL) made at the USFCMS-PL. Silica gel (high purity grade, 100–200 mesh, pore size 30A, Sigma Aldrich, USA) was combusted (450°C for 4h) and deactivated (2%) previous to column preparation for SPE. Biomarkers were collected using hexane (100%). All solvents used were the highest purity available. Two blanks were included in each set of samples (15–18 samples) to ensure no contamination during sample preparation. Biomarkers were quantified using GC/MS/MS multiple reaction monitoring (MRM) on a Varian 320 triple quadrupole MS. Splitless injections of 1μL of the sample were conducted. We used a RXi5sil column (30 m x 0.25 mm x 0.25 μm) with a GC oven temperature programming of 80°C held for 1 min, then increased to 200°C at a rate of 40°C/min, to 250°C at 5°C/min, to 300°C at 2°C/min, to 320°C at 10°C/min, and held for 2 min. The GC was operated in constant-flow mode (1ml/min) with an inlet temperature of 275°C and a transfer line temperature of 320°C. Ion source temperature was 180°C and source electron energy was 70eV. Argon at a pressure of 1 millitorr was used as a collision gas. We targeted biomarker compounds (hopanes, steranes, diasteranes) as conservative tracers for crude oil [49, 50]. Total concentration of biomarkers was calculated using the response factor by comparison with a known standard mixture (Calibration mix, Chiron, S-4436-10-IO) and the internal standard (d_4_-cholestane). When no commercial reference standard was available, compounds were quantified using the response factor for the nearest available homologue in the same compound class. Concentrations were corrected for the recovery of the surrogate standard (d_50_-Tetracosane). Recoveries from spiked samples included with each batch were generally within 60–80%. Replicate analyses were performed on selected samples and relative standard deviations (RSDs) of replicates (N = 4) for biomarker analysis were between 4% and 22%. Total biomarker concentration is expressed as sediment dry weight.

### Short-lived radioisotopes

Short-lived radioisotope analyses were conducted on all cores collected at the eleven sites and throughout the two-year time series. Samples were analyzed by gamma spectrometry on Series HPGe (High-Purity Germanium) Coaxial Planar Photon Detectors for total ^210^Pb (46.5Kev), ^214^Pb (295 Kev and 351 Kev), ^214^Bi (609Kev), ^137^Cs (661Kev), ^7^Be (447 Kev), and ^234^Th (63 Kev) activities. Data were corrected for counting time and detector efficiency, as well as for the fraction of the total radioisotope measured yielding activity in dpm/g (disintegrations per minute per gram).

Detector efficiencies were all <3% of the activities measured, determined by similar methods to Kitto [51]. The IAEA-447 organic standard, which has a similar density to the sediment analyzed in this study, was analyzed using varying weights (1g, 3g, 5g, 7g, 9g, 12g, 15g, 17g, 20g, 30g, 40g and 50g) as a proxy for geometry. A calibration template was produced relating the counts measured to the known activity of the standard for the range of sample weights. By using the calibration template for various weights, self-absorption of the sample is included in the detector efficiency calculations [52]. The Cutshall method [53] was used on select sediment samples, and results show that the self-absorption and variability is negligible and within detection error. The activity of the ^214^Pb (295 Kev), ^214^Pb (351 Kev), and ^214^Bi (609 Kev) were averaged as a proxy for the ^226^Ra activity of the sample or the supported ^210^Pb that is produced *in situ*. The supported ^210^Pb was subtracted from the total ^210^Pb to determine the unsupported (i.e., excess) ^210^Pb, which is used for dating within the last ~100 years [54]. ^137^Cs is a thermonuclear byproduct and represents the height of nuclear bomb testing in the early-mid 1960s [55], or other thermonuclear incidents [56]. ^7^Be has a short half-life (~53 days) and is an indicator of recent sediment deposition. ^234^Th has a half-life of ~24 days and is usually only detectable at the sediment surface. Supported ^234^Th was determined by reanalysis of the same sample >120 (~5 half-lives) days after core collection (i.e., all excess ^234^Th decayed). The supported ^234^Th was subtracted from the total ^234^Th to determine the unsupported (i.e., excess) ^234^Th. Activities of excess ^234^Th were corrected for activity decayed between the time of core collection and sample analysis and are termed “Decay Corrected ^234^Th”. Although excess ^234^Th is typically used as in indicator of surface mixing (e.g., bioturbation) [30, 57, 58], it has been used as a geochronological tool where sediments are unmixed [59].

In order to assign specific ages to sedimentary layers down core, excess ^210^Pb data were run through the CIC (Constant Initial Concentration) and CRS (Constant Rate of Supply) models, the latter of which is appropriate under conditions of varying accumulation rates [60, 61]. Activity values vs. depth down core were plotted for each core, and model results applied to assign a date to each individual sample. Mass accumulation rates (MAR) were calculated for each data point (i.e., “date”), thereby giving MAR over the past ~100 years. The use of mass accumulation rates corrects for differential sediment compaction down core, thereby enabling a direct comparison of excess ^210^Pb accumulation rates throughout the core (i.e., over the last ~100 years). Mass accumulation rates were calculated as follows:

$$MAR,g/{cm}^{2}/yr=dry\;bulk\;density\;(\rho )\times LAR$$

$$\text{Where}:\;dry\;bulk\;density,{g/cm}^{3}=dry\;weight÷sample\;volume$$

samplevolume=sampleinterval(z)×areaofcorebarrel(innerdiameter)

LAR=linearaccumulationrate,cm/yr

Recognizing that excess ^234^Th profiles may represent deposition and not bioturbation, excess ^234^Th-based MAR were calculated from CIC and CRS model results in the same fashion as excess ^210^Pb, as well as by simply dividing the depth of the excess ^234^Th penetration by 120 days (~5 half lives) to acquire LAR. MAR were then calculated according to the same equation as described above.

Sediment inventories of excess ^234^Th were calculated according to the method described in Baskaran and Santschi [62] following the equation:

$$I=(\rho_{i},A_{i},z_{i})$$

Where *I* is the excess ^234^Th inventory (dpm/cm^2^), *p*_*i*_ is the dry bulk density (g/cm^3^), *A*_*i*_ is the activity of excess ^234^Th (dpm/g) of sample *I*, and *z*_*i*_ is the thickness of sample *i* in cm. All excess ^234^Th inventories were decay corrected to the date of collection. Sediment inventories are independent of excess ^234^Th depth and therefore not impacted by bioturbation.

## Results

**Fig 2:**
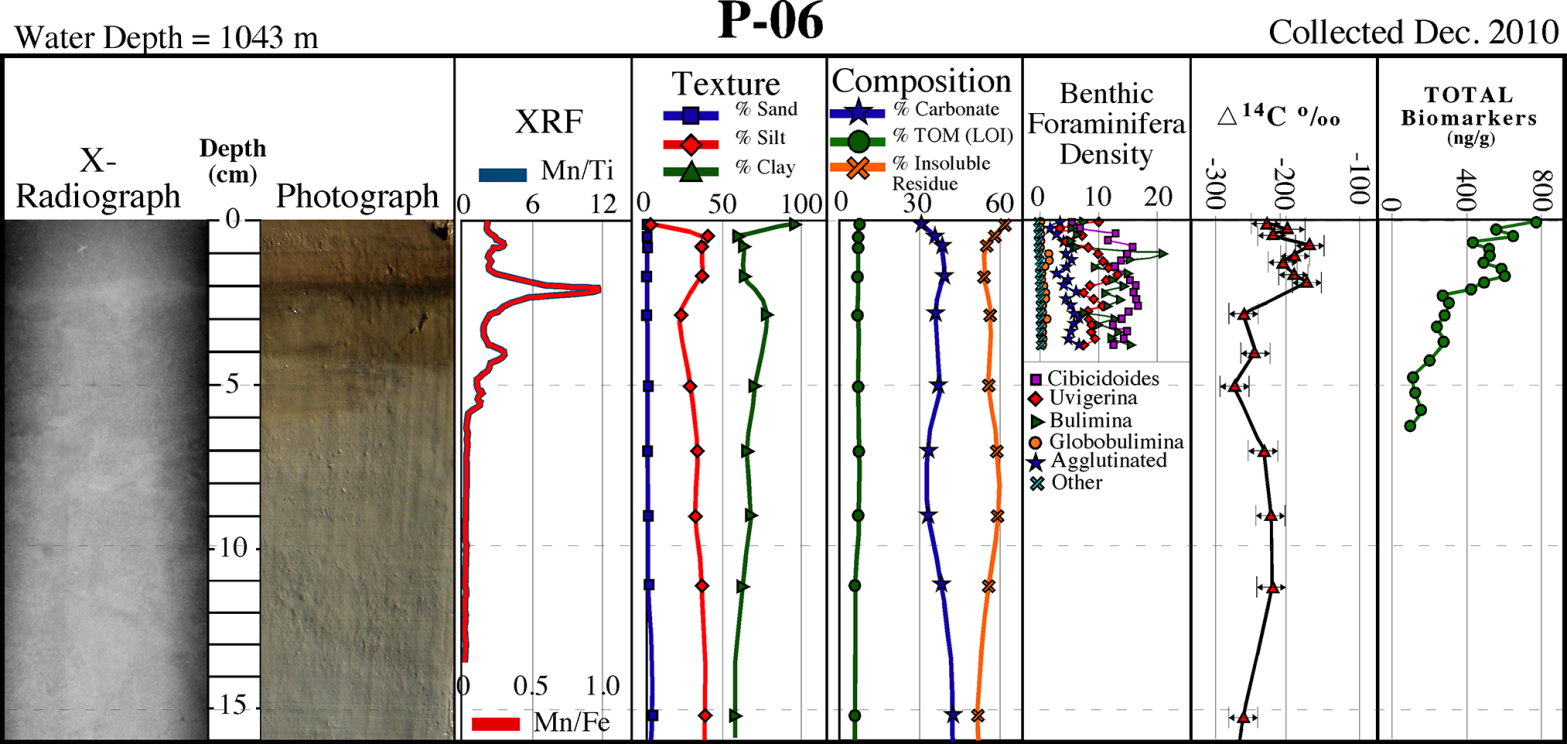
Core P-06 description. Description of core P–06 collected in December 2010 showing a surficial brown layer containing multiple dark brown-black bands corresponding to Mn spikes, and distinct sediment texture/composition, benthic foraminifera density, natural abundance radiocarbon (∆^14^C), and biomarkers over the surficial ~1 cm (see Fig 1 for location).

**Fig 3:**
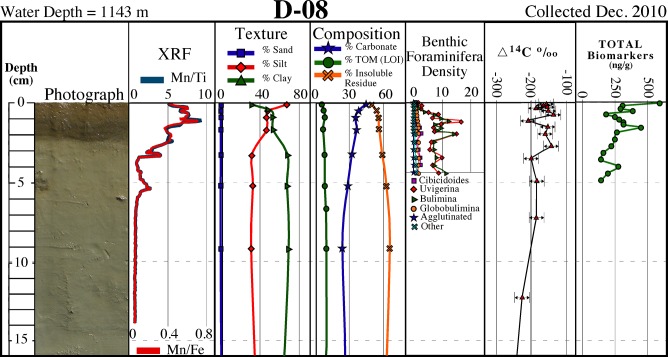
Core D-08 description. Description of core D–08 collected in December 2010 showing a surficial brown layer containing dark brown-black bands corresponding to Mn spikes, and distinct sediment texture/composition, benthic foraminifera density, natural abundance radiocarbon (∆^14^C), and biomarkers over the surficial ~1 cm (see Fig 1 for location).

**Fig 4:**
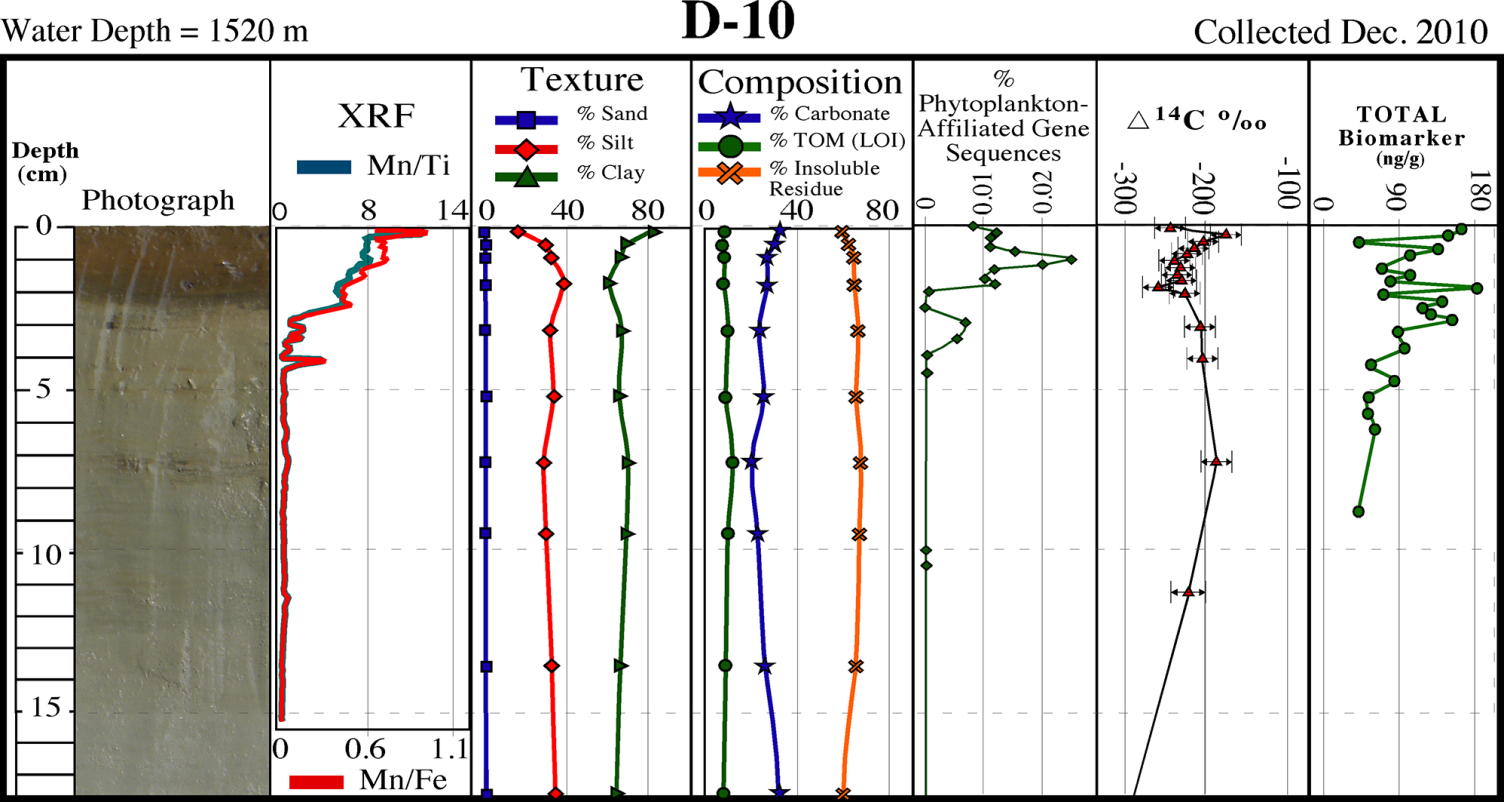
Core D-10 description. Description of core D–10 collected in December 2010 showing a surficial brown layer containing dark brown-black bands corresponding to Mn spikes, and a distinct sediment texture/composition, phytoplankton-affiliated gene sequences, natural abundance radiocarbon (∆^14^C), and biomarkers over the surficial ~1 cm (see Fig 1 for location).

With the exception of the shallowest core at ~100 m (M-01), the surficial ~1–10 cm of all cores collected were brown in color, overlying a massive light tan unit (Figs 2–4). The surface discoloration was typically thicker and better defined with increasing water depth. In most cores the medium to dark brown layer contained one or more ≤1 cm-thick dark brown-black bands that correspond with Mn spikes in XRF data (discussed below). Core photographs and x-radiographs show little in the way of sedimentary structures, although sand-sized biogenic particles (planktonic foraminifera and/or pteropods) were occasionally visible.

### Sediment texture and composition

Texturally, the grain size of all 2010 cores tends to become finer with increasing water depth as expected, and with few exceptions tends to fine-upward over the ~1 cm-thick surficial layers (Figs 2–4; Table 1). For cores collected in water depths of ≤600 m, the fining-upward unit is often manifested as a decrease in sand-sized sediments, whereas for sites in >1000 m depths it is often represented as an increase in clay-sized sediments. Carbonate content tends to increase slightly over the surface layer in cores collected in ≥600 m water depths (Figs 2–4; Table 1 **).** Both deep (>1000 m) sites on the siliciclastic-dominated west side of DeSoto Canyon (D-08 and D-10) recorded slight increases in carbonate content. Total organic matter (TOM) ranges from ~3% to ~12% with the highest percentages occurring in sediments from the deepest sites (Figs 2–4; Table 1). Down-core TOM percentages exhibit little variability (Figs 2–4; Table 1).

**Table 1:** Sediment texture and composition data at 2 mm intervals to excess ^234^Th depth, as well as average and ranges below excess ^234^Th depths to depths of excess ^210^Pb (~100 yrs). Data for cores D-08, D-10, and P-06 are shown graphically in Figs 2–4 and are not included here.

Site ID	Top Depth (cm)	Bottom Depth (cm)	% Gravel	% Sand	% Silt	% Clay	% Carbonate	% TOM (LOI)
	0.0	0.2	0.0	11.4	73.4	15.3	77.2	3.8
**M-01**	0.2	0.4	0.0	19.6	67.2	13.2	76.1	3.4
	0.4	0.6	0.0	19.5	77.3	3.1	76.4	3.2
	0.6	0.8	0.0	20.7	67.6	11.7	76.1	3.4
	0.8	1.0	0.0	17.0	68.8	14.2	76.4	3.1
Average	2.0	11.5	0.1	19.5	71.1	9.3	75.1	3.6
Range	2.0	11.5	0.0–0.4	16.3–24.6	67.8–75.5	4.5–14.2	74.4–75.9	3.1–4.1
	0.0	0.2	0.0	7.2	18.4	74.4	62.7	6.1
**M-03**	0.2	0.4	0.0	4.8	66.3	28.8	64.2	5.0
	0.4	0.6	2.2	11.1	64.8	21.9	61.6	6.8
Average	0.6	15.5	0.2	9.4	60.0	30.5	62.3	5.1
Range	0.6	15.5	0.0–0.6	3.0–15.7	49.9–79.4	12.2–40.0	58.5–65.3	3.2–6.4
	0.0	0.2	0.0	5.8	57.5	36.7	54.2	6.5
**M-04**	0.2	0.4	0.0	7.8	67.3	24.9	*NA	*NA
	0.4	0.6	0.0	13.9	64.2	21.9	59.1	6.6
	0.6	0.8	0.0	9.2	59.0	31.8	*NA	*NA
	0.8	1.0	0.0	5.5	65.4	29.1	54.7	6.8
	1.0	1.2	0.0	9.2	62.7	28.1	54.9	6.4
Average	1.2	16.5	0.0	20.4	49.9	29.7	57.4	6.4
Range	1.2	16.5	0.0–0.0	11.8–28.1	42.7–58.7	24.6–36.2	54.3–61.9	5.1–7.6
	0.0	0.2	0.0	0.8	46.0	53.3	54.1	6.7
**M-05**	0.2	0.4	0.0	0.6	38.8	60.6	42.6	8.4
	0.4	0.6	0.0	3.1	45.0	51.9	46.2	7.4
Average	0.6	9.5	0.0	7.8	48.2	44.0	46.9	7.7
Range	0.6	9.5	0.0–0.0	3.5–11.4	36.4–74.9	18.5–58.8	43.6–50.5	6.2–9.1
	0.0	0.2	0.0	0.6	49.3	50.1	55.0	4.7
**M-06**	0.2	0.4	0.0	0.8	58.7	40.4	52.2	6.2
	0.4	0.6	0.0	2.5	15.8	81.7	46.3	7.9
Average	0.6	**10.5**	0.0	4.1	45.1	50.8	44.9	7.0
Range	0.6	10.5	0.0–0.1	1.6–9.1	37.0–65.3	29.6–60.1	40.5–50.3	5.6–9.1
	0.0	0.2	0.0	16.4	44.9	38.7	62.2	5.4
**M-07**	0.2	0.4	0.0	23.2	48.5	28.3	66.5	4.6
	0.4	0.6	0.0	30.6	46.0	23.4	65.0	5.1
	0.6	0.8	0.0	30.1	44.5	25.4	68.6	4.3
	0.8	1.0	0.0	33.8	44.6	21.6	69.6	4.2
Average	1.0	15.5	0.0	26.9	45.0	28.1	64.1	4.7
Range	1.0	15.5	0.0–0.1	13.1–42.5	34.9–56.4	13.3–36.1	60.1–67.5	4.4–4.9
	0.0	0.2	0.0	49.8	30.7	19.5	73.2	3.7
**M-08**	0.2	0.4	0.0	47.5	35.3	17.2	73.3	4.8
	0.4	0.6	0.0	51.4	36.9	11.7	72.4	6.4
	0.6	0.8	0.0	53.3	31.1	15.6	74.6	5.0
Average	0.8	17.5	0.1	45.0	34.6	20.2	71.0	5.0
Range	0.8	17.5	0.0–0.8	29.8–65.9	27.1–49.8	5.0–30.2	51.9–79.0	2.9–8.1
	0.0	0.2	0.0	2.5	60.0	37.4	59.7	6.0
**M-09**	0.2	0.4	0.0	4.8	72.1	23.1	*NA	*NA
	0.4	0.6	0.0	7.9	61.7	30.4	*NA	*NA
Average	0.6	16.5	0.0	11.4	48.4	40.2	54.9	6.0
Range	0.6	16.5	0.0–0.2	6.0–18.9	39.0–65.0	25.0–48.5	53.1–57.8	5.5–6.9

*NA–Not Analyzed

Microscopic analyses for three 2010 time series sites (D–08, D–10, P–06) using both the digital microscope (70x-200x) and SEM (2kx-8kx magnification) show that sediments consist predominantly of unidentifiable, amorphous aggregates with trace amounts of identifiable siliciclastic grains and biogenic carbonates. SEM analysis showed biogenic material to consist predominantly of coccolithophore plates, which appeared to be more common near the sediment surface. Otherwise, no discernible difference(s) were evident between the surface and underlying layers in the three cores analyzed.

Elemental composition, determined by SEM/EDS and Scanning XRF, yielded similar results in that, with few exceptions, no discernable differences were evident between the surface and underlying layers. EDS data showed surficial and underlying sediments from all 2010 cores analyzed (D–08, D–10, P–06) to consist dominantly of Si, O, Al, and Ca with subordinate amounts of C, Mg and K. Scanning XRF data for the same three cores showed no appreciable differences in lithogenic elements (Ti, Al, Fe, Si) and/or biogenic elements (Ca, Si) between the surface and underlying layers. An exception is Mn, which substantially increased in the surficial ~1–10 cm brown layer, and consistently exhibited pronounced spikes correlating to the ≤1 cm-thick darkest brown-black bands that occur within this interval (Figs 2–4).

XRD results showed a detrital silicate and biogenic carbonate mineral assemblage considered typical for the NE GoM. Dominant clay minerals include smectite and kaolinite, as expected. No discernable variations in mineralogical composition over the surficial layer, as compared to down-core, was evident.

### Natural abundance radiocarbon

Natural abundance radiocarbon, analyzed on 2010 time series cores P-06, D-08 and D-10 (Figs 2–4), exhibited a reproducibility of ±6.5‰ based on 17 replicate samples. We hypothesized that if significant quantities of petroleum-based carbon had been input to surface layers, then surficial sediments would be depleted in ^14^C relative to underlying sediments, as observed at sites P-06 and D-10, the sites closest to the DWH wellhead (Fig 1). Most petro-carbon depletion was to the south and west of the wellhead, although some migrated to the northeast also [63]. Consistent with our observations, Chanton et al. [63], and Valentine et al. [23], observed that petro-carbon deposition was mainly within the 0–1 cm surface interval of sediments.

### Microbial community structure

Microbial communities were characterized using next generation sequencing of SSU rRNA gene sequences. Overall, communities were dominated by members of the prokaryotic phyla Proteobacteria, Planctomycetes, Chloroflexi, and Thaumarchaeota. These phyla were observed at high relative abundance in all cores sampled in the northern Gulf and likely represent the core community observed in sediments of this region. Since chloroplasts of eukaryotes also contain rRNA genes, eukaryotic algae may also be detected in our dataset. Unlike other microbial groups mentioned above, sequences affiliated photosynthetic microbial groups, Cyanobacteria (Synechococcus) and chloroplasts of marine diatoms, were significantly enriched by one order of magnitude (p < 0.00008) in surficial (0–2 cm depth) sediments compared to underlying sections (Fig 4). The relative abundances of these planktonic phototroph sequences reached a maximum at ~1 cm sediment depth. Sequences related to the Bacillariophyta comprised the majority of detected phytoplankton chloroplast sequences (> 98%).

### Benthic foraminifera

A decline in benthic foraminiferal density was evident in all 2010 time series cores analyzed. This decline is represented by a continuous decrease below down-core means of 80–93% in assemblage density (all genera, infaunal and epifaunal) and benthic foraminiferal accumulation rate (BFAR) in the surficial ~1 cm in cores P-06 and D-08 (Figs 2 and 3).

### Biomarkers

All 2010 time series cores analyzed for total biomarkers showed elevated concentrations over the surface ~1 cm (Figs 2–4). A comparison of the ~1 cm thick surface interval to underlying sediments indicated an increase in the concentration of total biomarkers in the surface sediment layer by 26% in D-10, 37% in D-08, and 72% in P-06.

### Short-lived radioisotopes

Excess ^210^Pb and ^234^Th were detected in almost all cores. When detected, ^137^Cs and ^7^Be levels were exceptionally low, which is consistent with other reports [30], and will not be discussed here. Excess ^210^Pb was detected in all eleven November/December 2010 cores to depths ranging from ~10–19 cm. Mass accumulation rates over the past ~100 years ranged from 0.05–0.16 g/cm^2^/yr (Table 2), which is consistent with rates previously reported for the NE GoM [30]. Excess ^234^Th was detected in all November/December 2010 cores, except for Core M-01, collected at the shallowest depth of 100 m (Fig 1). Excess ^234^Th depths ranged from 0.4 to 1.2 cm (Table 2). Excess ^234^Th-based MAR calculated by the CRS model are reported here (because they are the most conservative) and range from 0.48 to 2.40 g/cm^2^/yr **(** Table 2). Sediment inventories of excess ^234^Th ranged from 0.37 to 2.72 dpm/cm^2^ (Table 2).

**Table 2:** Excess ^234^Th and excess ^210^Pb profile depths, MAR, and inventories.

Site ID			Nov. 2010	Dec. 2010	Feb. 2011	Sept 2011	Aug. 2012	Oct. 2012
**D-08**		Depth (cm)		1.2		0.4		
	^234^Th	MAR (g/cm^2^/yr)		2.00*		0.40+		
1143		Inventory (dpm/cm^2^)		1.04*		0.19+		
	^210^Pb	Depth (cm)		13.5				
		MAR (g/cm^2^/yr)		0.07				
**D-10**		Depth (cm)		0.4	0.4	0.4	0.4	
	^234^Th	MAR (g/cm^2^/yr)		0.60*	0.48*	0.25+	0.14+	
1520		Inventory (dpm/cm^2^)		1.68*	1.70*	0.40+	0.26+	
	^210^Pb	Depth (cm)		18.0				
		MAR (g/cm^2^/yr)		0.12				
**P-06**		Depth (cm)		1.2		0.4	0.4	
	^234^Th	MAR (g/cm^2^/yr)		1.35*		0.35+	0.66+	
1043		Inventory (dpm/cm^2^)		1.06*		0.06+	0.35+	
	^210^Pb	Depth (cm)		16.0				
		MAR (g/cm^2^/yr)		0.06				
**M-04**	^234^Th	Depth (cm)	1.2					0.4
	^234^Th	MAR (g/cm^2^/yr)	1.76*					0.20+
400		Inventory (dpm/cm^2^)	1.34*					0.44+
	^210^Pb	Depth (cm)	16.0					
		MAR (g/cm^2^/yr)	0.07					
**M-01**	^234^Th	Not Detected						
100	^210^Pb	Depth (cm)	10.0					
		MAR (g/cm^2^/yr)	0.16					
**M-03**		Depth (cm)	0.6					
	^234^Th	MAR (g/cm^2^/yr)	1.09*					
300		Inventory (dpm/cm^2^)	1.89*					
	^210^Pb	Depth (cm)	16.0					
		MAR (g/cm^2^/yr)	0.07					
**M-05**		Depth (cm)	0.6					
	^234^Th	MAR (g/cm^2^/yr)	1.17*					
500		Inventory (dpm/cm^2^)	1.01*					
	^210^Pb	Depth (cm)	9.0					
		MAR (g/cm^2^/yr)	0.08					
**M-06**		Depth (cm)	0.6					
	^34^Th	MAR (g/cm^2^/yr)	2.4*					
600		Inventory (dpm/cm^2^)	0.59*					
	^210^Pb	Depth (cm)	10.0					
		MAR (g/cm^2^/yr)	0.05					
**M-07**		Depth (cm)	1.0					
	^234^Th	MAR (g/cm^2^/yr)	1.34*					
400		Inventory (dpm/cm^2^)	1.24*					
	^210^Pb	Depth (cm)	15.0					
		MAR (g/cm^2^/yr)	0.07					
**M-08**		Depth (cm)	0.8					
	^234^Th	MAR (g/cm^2^/yr)	1.58*					
400		Inventory (dpm/cm^2^)	2.72*					
	^210^Pb	Depth (cm)	18.0					
		MAR (g/cm^2^/yr)	0.15					
**M-09**		Depth (cm)	0.6					
	^234^Th	MAR (g/cm^2^/yr)	0.74*					
400		Inventory (dpm/cm^2^)	0.37*					
	^210^Pb	Depth (cm)	16.5					
		MAR (g/cm^2^/yr)	0.07					

* denotes Late 2010/Early 2011 values in Table 3

^+^ denotes Late 2011/2012 values in Table 3.

**Fig 5:**
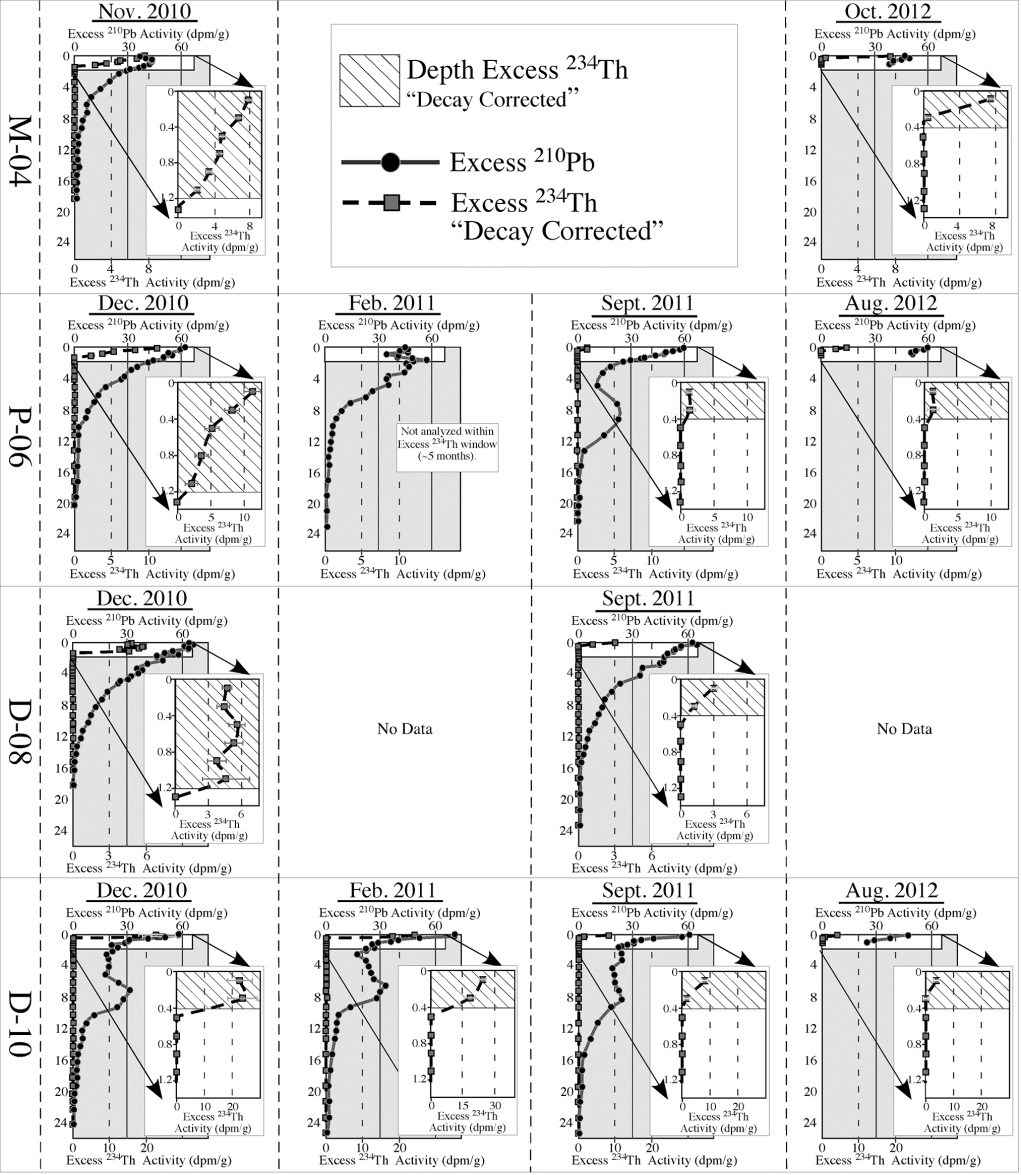
Excess ^210^Pb and ^234^Th profiles for time series sites. Excess ^210^Pb and excess ^234^Th profiles for time series cores collected at site M-04 in November 2010 and October 2012, and sites P–06, D–08, and D–10 collected in December 2010, February 2011, September 2011 and August 2012. Profiles are expanded to show the decrease in decay-corrected excess ^234^Th activities and excess ^234^Th depths following initial coring in December 2010 (see Fig 1 for core site locations).

**Fig 6:**
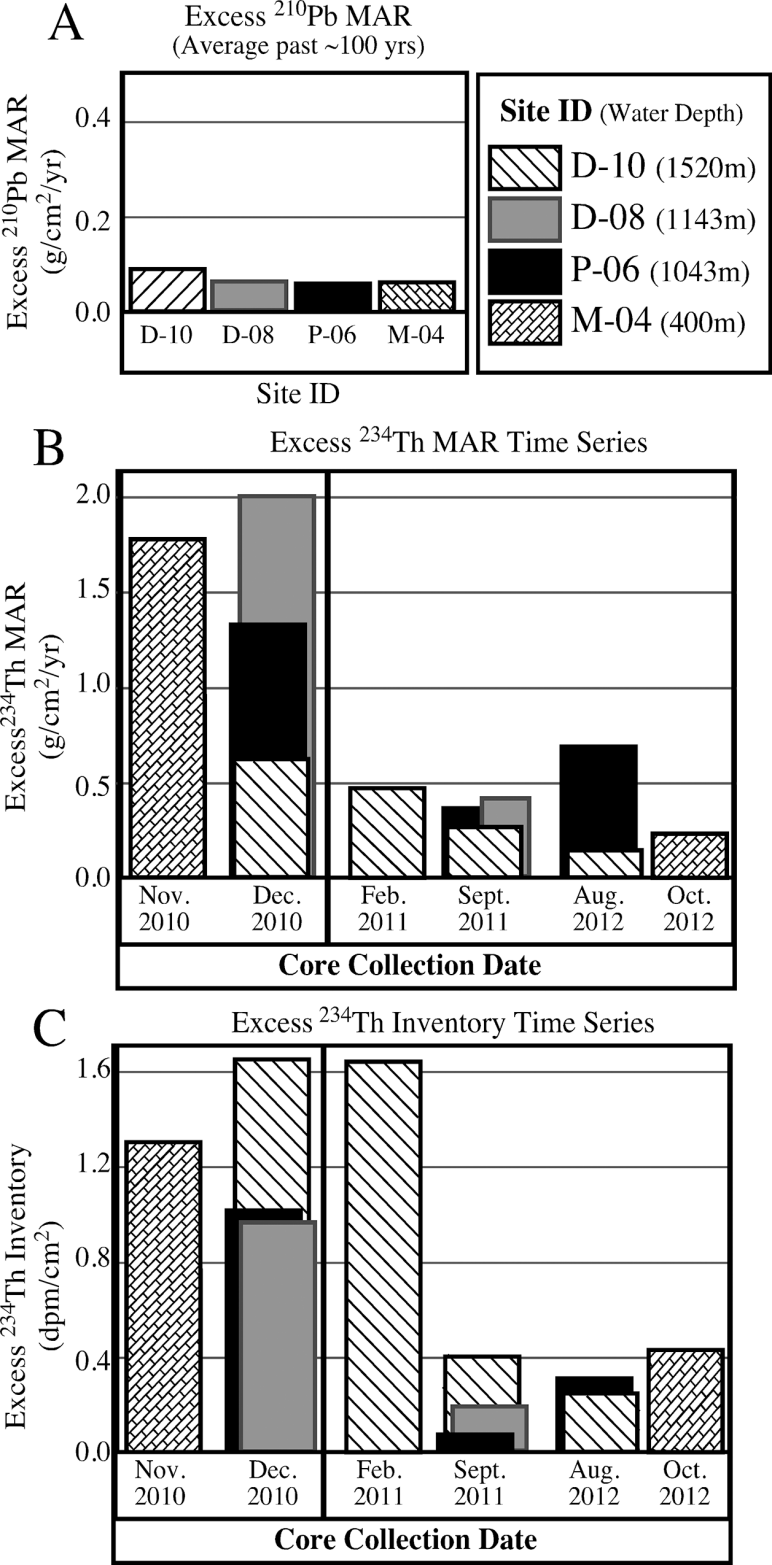
Mass accumulation rates (MAR) and ^234^Th inventories for time series sites. Graphs showing (A) average MAR over the past ~100 years calculated using excess ^210^Pb, (B) MAR of the four time series sites from November 2010 to October 2012 calculated using excess ^234^Th, (C) excess ^234^Th inventories of the four time series sites from November 2010 to October 2012.

Excess ^210^Pb and ^234^Th was detected in all cores collected from the four time series sites (M-04, D-08, D-10, P-06) over the entire two-year period (Table 2). Excess ^234^Th profile depths, MAR, and inventories are all highest in cores collected in late 2010/early 2011, after which they decreased rapidly (within a few to several months) and then remain relatively stable over the following ~2 years **(**Figs 5 and 6; Table 2 **)**. Excess ^234^Th inventories and MAR were categorized by their core collection date. Late 2010/early 2011 inventories and MAR were compared to late 2011 and 2012 inventories and MAR with a student’s T test and differences between the two time periods were highly significant, p = 0.007 for MAR and p = 0.0018 for inventories (Table 3).

**Table 3:** Results of T–test using Excess ^234^Th MAR and Inventories from *Late 2010/Early 2011 time period and ^+^Late 2011/2012 time period.

	* Late 2010/ Early 2011 MAR	^+^Late 2011/2012 MAR	* Late 2010/ Early 2011 Inventory	^+^Late 2011/2012 Inventory
	1.76	0.40	1.34	0.19
	1.09	0.25	1.89	0.40
	1.17	0.35	1.01	0.06
	2.40	0.14	0.59	0.26
	1.34	0.66	1.24	0.35
	1.58	0.20	2.72	0.44
	0.74		0.37	
	2.00		1.04	
	0.60		1.68	
	1.30		1.06	
	0.48		1.70	
Average	1.31	0.33	1.34	0.28
Standard Deviation	0.59	0.19	0.66	0.14
Standard Error	0.18	0.08	0.20	0.06
n	11	6	11	6
**P Value**	**0.0070**		**0.0018**	

## Discussion

Multiple independent lines of sedimentological, geochronological, geochemical, and biological evidence point to a rapid, but short-lived sedimentation event from surface waters to the deep sea floor of the NE Gulf of Mexico (GoM) in late 2010 that was coincident with the formation of oil slicks and marine snow formation from the DWH discharge [16, 23, 63]. A radionuclide distribution time series from sites occupied multiple times between 2010 and 2012 indicates greater excess ^234^Th depths, MAR and inventories in late 2010/early 2011 relative to later collection periods (Figs 5 and 6). Consistent dissimilarities in grain size and natural abundance radiocarbon over the surficial ~1 cm of sediments relative to underlying sediments are consistent with a lack of downward mixing of the surface ~1 cm into underlying, relatively homogeneous sediments by bioturbation, or other processes. Stratification in the form of speciation of metals, specifically solid phase Mn, provides evidence for redox change in sediments that is consistent with elevated sedimentation rates [64]. Total biomarker concentrations in sediments were also elevated above baseline levels (Figs 3 and 4). A dramatic decrease in benthic foraminifera density in surficial sediments coincided with the lack of bioturbation, apparent elevated mass accumulation rates and total biomarker concentrations (Figs 3 and 4). Lastly, a substantial enrichment in SSU rRNA gene sequences derived from photosynthetic organisms (phytoplankton chloroplasts) that normally occupy the sea surface mixed layer were detected in the top ~2 cm of deep-sea sediment cores, which is consistent with the hypothesis of a depositional event directly after the Deepwater Horizon (DWH) discharge (Fig 4). Both the microbial community structure (relative abundance of phytoplankton-affiliated gene sequences) and hydrocarbon chemistry (recalcitrant biomarkers) suggest input/deposition of material from surface waters was recorded in surficial sediments of cores collected in December, 2010.

Sedimentological, biological and chemical evidence are consistent with the rapid deposition of a layer corresponding to the depth of excess ^234^Th. In the absence of downward mixing, we hypothesize that the observed excess ^234^Th profiles reflect deposition, and that the entire 0.4–1.2 cm thick surface layer was deposited rapidly, within a period of 4–5 months. A central issue that must be addressed is that although the down-core excess ^234^Th profiles are consistent with decay profiles, bioturbation could produce similar distributions [65, 66]. Little information is available on bioturbation in shelf and slope sediments of the Gulf of Mexico. Polychaetes (38%) and amphipods (21%) comprised the majority of the macrofaunal (primary bioturbators) standing stock along the north-central and northeastern GoM slope previous to the DWH event [67]. Anecdotally, during foraminiferal identification [68], there were no visible skeletal remains of polychaete or amphipod taxa in the surface 50 mm of the D-08 and P-06 cores collected in December 2010 and February 2011. Bioturbation depths reported for deep-sea sediments in the GoM at sites near our study area (1.75 to 3.25 cm) are larger than the maximum excess ^234^Th depths (1.2 cm) observed in this study [30]. However, the variation in excess ^234^Th inventory is consistent with increased sediment deposition in 2010 and early 2011, and cannot be explained by variations in bioturbation. Bioturbation could increase the excess ^234^Th depth, but would not affect the excess ^234^Th inventory.

At steady state, excess ^234^Th inventories should be directly proportional to sediment accumulation rates. Under steady state conditions, the flux of excess ^234^Th to the seafloor (*J* in dpm cm^-2^ y^-1^) is directly proportional to the inventory of excess ^234^Th in seafloor sediments (*I* in dpm cm^-2^) multiplied by the decay constant (in y^-1^):

J=λ×I

The summed decay rate of excess ^234^Th in the sediments is equal to the decay constant (**λ**) multiplied by the excess ^234^Th inventory (*I)* in sediments. At steady state (constant inventory) this decay rate is balanced by the input of new excess ^234^Th. As ^234^Th is highly particle reactive, excess ^234^Th input should be directly proportional to the sediment accumulation rate, and the input rate of excess ^234^Th should balance the decay rate. Since the decay rate is directly proportional to the inventory, so is the input rate and thus the sediment accumulation rate, assuming relatively steady state conditions on the time scale of the life of the tracer, which is several months.

Our results demonstrate that excess ^234^Th inventories decreased by a factor of 4–5 from late 2010 to late 2011 and 2012. Thus we assert that sediment accumulation rates follow the same trend. Excess ^234^Th inventories are independent of bioturbation, which would merely redistribute excess ^234^Th, not change the quantity of it. Over time, we observe the excess ^234^Th inventory decrease, which is consistent with decreasing rates of excess ^234^Th input via decreased sedimentation rates. Excess ^234^Th-derived sediment mass accumulation rates were at least 4 times higher in late 2010 (0.48 to 2.40 g.cm^-2^y^-1^), as compared to 2011 and 2012 (0.14 to 0.66 g cm^-2^y^-1^) (Figs 5 and 6).

The dramatic decline in excess ^234^Th depth, mass accumulation rates (MAR), and excess ^234^Th inventories (which is independent of bioturbation) in our time series results (Figs 5 and 6; Table 2) are consistent with the occurrence of a brief, but rapid depositional event in summer/fall 2010 after the DWH discharge. These results indicate that the depositional event quickly subsided in 2011, and sedimentation remained relatively constant over the subsequent two years (Figs 5 and 6). The depositional pulse was detected in continental slope sediments between ~300 m and ~1500 m both to the east and west of DeSoto Canyon. The 100 m site (M-01) revealed no indication of a distinct surface layer and no excess ^234^Th signal. Although excess ^234^Th-derived MAR for the surface layer are considerably higher than average rates calculated for the previous ~100 years using excess ^210^Pb (Table 2), rates determined using these different methods cannot be directly compared due to the differences in time scales involved [69, 70].

In contrast to surface sediment layers characterized in previous work conducted closer to the wellhead [17, 19], the distinct color change in the surface layer (<1–10 cm) of this study is not a reflection of the sedimentology or petroleum input, but reflects diagenetic processes that are consistent with a rapid depositional event. The ≤1 cm-thick dark brown-black color bands within the surficial brown layer represent spikes in manganese (Mn) oxides, as indicated by enrichments of Mn relative to titanium (Ti) and iron (Fe) in XRF core scans (Figs 3 and 4). Manganese oxide enrichments are commonly observed in pelagic surface sediments due to redox-related cycling of Mn in association with organic matter diagenesis. Below the oxygen penetration depth in the sediment column, Mn oxides are utilized as electron acceptors in ongoing organic matter remineralization [71]. This process releases dissolved Mn^2+^ into pore waters, which then diffuses vertically upwards and reprecipitates as Mn oxides upon contact with dissolved oxygen. In pore waters, such Mn cycling results in a single, well-defined peak of Mn oxide close to the oxygen penetration depth [72]. However, most cores analyzed in this study show multiple Mn peaks in the upper sediments, suggesting a pulsing of sediment input. Multiple Mn peaks in sediment cores have been interpreted to indicate vertical shifts in the oxygen penetration depth [73, 74], which causes the active Mn peak to shift vertically, leaving a relict peak at the former position. Bulk Mn sampled at mm-scale resolution, digested in strong acid, and measured by ICP-MS by Hastings [64] corroborates the multiple Mn peaks we observe and the rapid shoaling of the Mn oxide peak.

### Depositional mechanisms

Our observations are consistent with a depositional pulse driven by the formation and rapid settling of the large marine snow particles, as documented in overlying surface waters of the northern GoM during early summer 2010 [14]. Elevated hopane concentrations in sediments [23, 75], depletion in natural abundance radiocarbon [63], and the detection of oil-associated marine snow [16], is consistent with the incorporation of DWH oil in marine snow particles and their rapid sedimentation to the deep NE GoM. Specifically, hopanes, steranes, and diasteranes, which are widely used for oil fingerprinting, detected in the surface sediment pulse layer of cores used in this study, indicated the presence of DWH oil. Sediments below the surface layer and from a control site (all depth intervals) showed no match with DWH oil [75]. Following the DWH event, marine snow aggregates over a wide range of size classes formed in surface oil slicks and possibly in subsurface oil plumes [7, 14]. Once buoyancy was lost, the marine snow rapidly settled to the sea floor. The detection of gene sequences affiliated with planktonic diatoms originating from sea surface habitats in the surficial sediment layer (Fig 4), as well as larger concentrations of petrogenic hydrocarbons (Figs 3 and 4), is consistent with rapid settling of sea-surface material to the sea floor. Upon reaching the sea floor, organic matter was respired, creating reducing conditions in the sediments [64], thus apparently inhibiting bioturbation and facilitating the preservation of the sediment pulse layer. The relatively consistent siliciclastic and biogenic sedimentary components in surface sediments suggests that sediment sources did not noticeably change, but the depositional mechanism created a much higher flux rate of the natural particles in the water column to the sea floor, as supported by sediment trap observations by Passow (Pers. Comm, 2013). The slight increase in carbonate content and coccolithophores observed in some surface sediments is consistent with the observed increase in the clay-size fraction of the surface interval in some deeper cores.

Although our findings are consistent with a documented marine snow event, alternative depositional mechanisms must be considered. For example, the intentional discharge of Mississippi River water to repel oiled waters from coastal regions [76] would be expected to increase siliciclastic input/deposition. The unusually high seasonal runoff may also have increased siliciclastic input/deposition. An increase in bio-mineral (i.e., carbonate and/or siliceous) production due to nutrient input from increased Mississippi River discharge may also have occurred. However, no significant increase in siliciclastic composition was detected. In fact, the only systematic variation in sediment composition was a subtle increase in calcium carbonate content in the two deep cores (D-08 and D-10) west of DeSoto Canyon, which is where increased siliciclastic input would be most expected. In addition, with one exception (core P-06), cores collected to the east of DeSoto Canyon, a carbonate province generally believed to receive little input from Mississippi River sediments, recorded the pulse with no increase in siliciclastic input. Additionally, Mississippi River discharge could not explain the elevated hopane, sterane, and diasterane concentrations [23, 75] and depletion in natural abundance radiocarbon [63] in NE GoM surface sediments that are indicative of petroleum hydrocarbon input. Thus, although input from the Mississippi River may have played a role, our evidence supports marine snow as the primary depositional mechanism.

Advective, or lateral sediment transport is another possibility, and is not uncommon in deep-sea settings [77], including the NE GoM [78]. Advection could explain the variations in excess ^234^Th inventories in our two-year time series due to sediment focusing. However, our observations of organisms and chemicals transferred from the sea-surface to surficial sediments (phytoplankton gene sequences and biomarkers) and the consistency in sediment source cannot be explained by an advective transport mechanism. Though advective transport undoubtedly plays a role in depositional patterns of the NE GoM and should continue to be investigated, our results are more consistent with a sedimentation pulse originating from the sea surface.

## Conclusions

A depositional pulse was recorded in bottom sediments in the DeSoto Canyon region of the NE Gulf of Mexico during late summer and fall of 2010, as a ~1 cm-thick sedimentary layer extending up to 100 nautical miles northeast of the DWH wellhead in water depths ranging from ~300 to ~1500 m. The sediment pulse layer was detected in two diverse sedimentological regimes, exhibited sedimentary properties distinctly different from underlying sediments, and included components originating from the sea surface. The depositional mechanism is interpreted to be an extensive marine snow event that was observed in surface waters over the study area during the summer of 2010. Independent studies have linked the marine snow event with the 2010 DWH blowout. Sediments below the surface pulse layer are generally homogeneous and contain no evidence of previous similar depositional events, which suggests that either this was a unique occurrence, or that deposits resulting from such events have not been preserved in the sedimentary record. Continued study will help to determine if/how this depositional event will eventually be recorded in bottom sediments in the NE GoM.
